# MERS-CoV accessory ORF proteins in innate immune evasion: an evidence-graded functional framework

**DOI:** 10.3389/fimmu.2026.1963823

**Published:** 2026-09-29

**Authors:** Chunhua Wang, Xuhang Li, Yi Jiang, Jinhui Yang, Minghui Yang, Yang Yang, Mengjun Tang, Wenjie Tan, Ji Zhang

**Affiliations:** 1 Department of Central Laboratory, Xiangyang Central Hospital, Affiliated Hospital of Hubei University of Arts and Science, Xiangyang, Hubei, China; 2 Department of Clinical Laboratory, Xiangyang Hospital of Traditional Chinese Medicine [Xiangyang Institute of Traditional Chinese Medicine], Xiangyang, Hubei, China; 3 School of Clinical Medicine, Hubei University of Arts and Science, Xiangyang, Hubei, China; 4 School of Life Science, Key Laboratory of Molecular Medicine and Biotherapy, Beijing Key Laboratory of Mucosal Immunity Vaccines Against Respiratory Viruses, Beijing Institute of Technology, Beijing, China; 5 National Clinical Research Center for Infectious Disease, State Key Discipline of Infectious Disease, Shenzhen Third People’s Hospital, Second Hospital Affiliated to Southern University of Science and Technology, Shenzhen, China; 6 NHC Key Laboratory of Biosafety, National Institute for Viral Disease Control and Prevention, Chinese Center for Disease Control and Prevention, Beijing, China

**Keywords:** accessory protein, innate immune evasion, MERS-CoV, ORF3, ORF4a, ORF4b, ORF5, ORF8b

## Abstract

Middle East respiratory syndrome coronavirus (MERS-CoV) is a merbecovirus associated with recurrent zoonotic spillover and severe lower-respiratory disease. Research has focused largely on DPP4-mediated entry, spike structure, replicase biology, neutralizing antibodies, and vaccines. However, the 3′-encoded accessory proteins are increasingly recognized as context-dependent modulators of viral fitness and pathogenesis. ORF4a binds double-stranded RNA (dsRNA) and suppresses MDA5- and PKR-dependent antiviral responses; ORF4b antagonizes OAS–RNase L and NF-κB signaling and contributes to inflammatory pathology in defined infection models; ORF5 modulates interferon and inflammatory responses in a genetic-background-dependent manner; and ORF8b interferes with MDA5- and HSP70–IKKϵ-dependent signaling, although its pathogenesis phenotype varies by experimental system and viral background. ORF3 shows HUWE1-regulated pro-apoptotic activity in ectopic-expression systems, but its role during authentic MERS-CoV infection remains unresolved. Our review organizes these findings by evidence type, pathway position, cell or tissue context, sampling time, and genetic-interaction evidence. This framework separates demonstrated mechanisms from inferred biological consequences and identifies the validation required before accessory-ORF biology can inform host-range assessment, therapeutic development, sequence surveillance, or rational attenuation.

## 1 Introduction

Since its identification in 2012, MERS-CoV has remained a high-priority zoonotic coronavirus because recurrent animal-to-human spillover can result in severe lower-respiratory disease. Initial virus isolation and genomic characterization established the etiological agent and its genome organization (1, 2), and subsequent studies identified DPP4/CD26 as the entry receptor and defined the spike–receptor interface (3, 4). Yet receptor compatibility is only the first barrier to productive infection. Viral fitness after entry also depends on how replication intersects with RNA sensing, interferon induction, translational arrest, RNA degradation, inflammatory signaling, and cell-death pathways.

MERS-CoV generally transmits less efficiently between humans than SARS-CoV or SARS-CoV-2, although healthcare-associated outbreaks show that substantial transmission can occur under favorable epidemiological conditions (5). Its accessory proteins ORF3, ORF4a, ORF4b, ORF5, and ORF8b have therefore been studied as modulators of host defense. Mechanistic support is strongest for discrete functions demonstrated in biochemical, cellular, recombinant-virus, and animal systems; support for direct cooperation among accessory proteins, cross-species adaptation, clinical effects, therapeutic tractability, and surveillance utility is less complete. Crucially, convergence on the same pathway does not establish redundancy, additivity, synergy, or hierarchical regulation.

Our review therefore organizes accessory-ORF biology along five axes: experimental strength, pathway position, cell or tissue context, sampling time, and genetic-interaction evidence. This approach separates mechanistic convergence from tested cooperation and distinguishes established functions from hypotheses concerning host-dependent fitness, therapeutic targeting, sequence-to-function surveillance, and rational attenuation.

## 2 Literature search and evidence appraisal

We conducted a targeted narrative literature search of PubMed/MEDLINE, PubMed Central, Web of Science Core Collection, Google Scholar, and relevant journal databases from database inception through 1 September 2026. Searches used combinations of “MERS-CoV” or “Middle East respiratory syndrome coronavirus” with “accessory protein,” “ORF3,” “ORF4a,” “ORF4b,” “ORF5,” “ORF8b,” “immune evasion,” “interferon,” “innate immunity,” “inflammation,” “pathogenesis,” “virulence,” “reverse genetics,” “animal model,” and “human infection.” Pathway-specific searches additionally used terms including “dsRNA,” “PKR,” “OAS,” “RNase L,” “nuclear transport,” “importin,” and “ubiquitin” when evaluating individual mechanisms.

We prioritized peer-reviewed primary studies that directly examined MERS-CoV accessory proteins in ectopic-expression systems, infected cells, recombinant viruses, animal models, human-derived samples, or structural and biochemical experiments. Reviews were used for background and citation tracking but not as primary evidence; non-peer-reviewed sources were not used as primary evidence. Findings were classified by experimental context as ectopic-expression, infected-cell, recombinant-virus, heterologous-virus, animal-model, human, or structural/biochemical evidence. Studies were included when they directly informed accessory-ORF function, host-response regulation, pathogenesis, or translational interpretation.

Throughout this Review, functional convergence denotes effects on the same or related host processes without implying genetic interaction. Cooperation denotes a joint contribution demonstrated by combined perturbation and implies neither additivity nor synergy. An additive effect matches a prespecified expectation derived from the corresponding single perturbations, whereas a synergistic effect exceeds that expectation. Redundancy requires evidence that loss of one factor is buffered or compensated by another. Hierarchical regulation requires directional dependence established by epistasis, temporal perturbation, or comparable causal analysis. Where these criteria are not met, we use functional convergence, combined effect, or working model. For consistency, ORF4a, ORF4b, and ORF5 are used throughout; NS4a, NS4b/4b, and gene 5 are retained only in bibliographic titles or when discussing source terminology.

## 3 Coding map, evolutionary origin, and research paradigms of MERS-CoV accessory proteins

### 3.1 Genomic organization of MERS-CoV and distribution of accessory open reading frames

The MERS-CoV genome follows the canonical coronavirus organization of a 5′ replicase region and a 3′ structural-accessory region. In the EMC/2012 reference genome (GenBank JX869059.2), ORF3 spans nt 25,532–25,843, ORF4a nt 25,852–26,181, ORF4b nt 26,093–26,833, and ORF5 nt 26,840–27,514; ORF4a and ORF4b therefore overlap by 89 nt. ORF8b spans nt 28,762–29,100 and is an internal ORF embedded within the N gene (nt 28,566–29,807) in an alternative reading frame (6) (**Figure 1**). Early ectopic-expression screening showed that M, ORF4a, ORF4b, and ORF5 can inhibit IFN-β promoter activity or downstream IRF3/7, NF-κB, and ISRE signaling to different degrees (7). These results established candidate immune-regulatory functions but do not, by themselves, demonstrate cooperation among the proteins during authentic infection.

**Figure 1:**
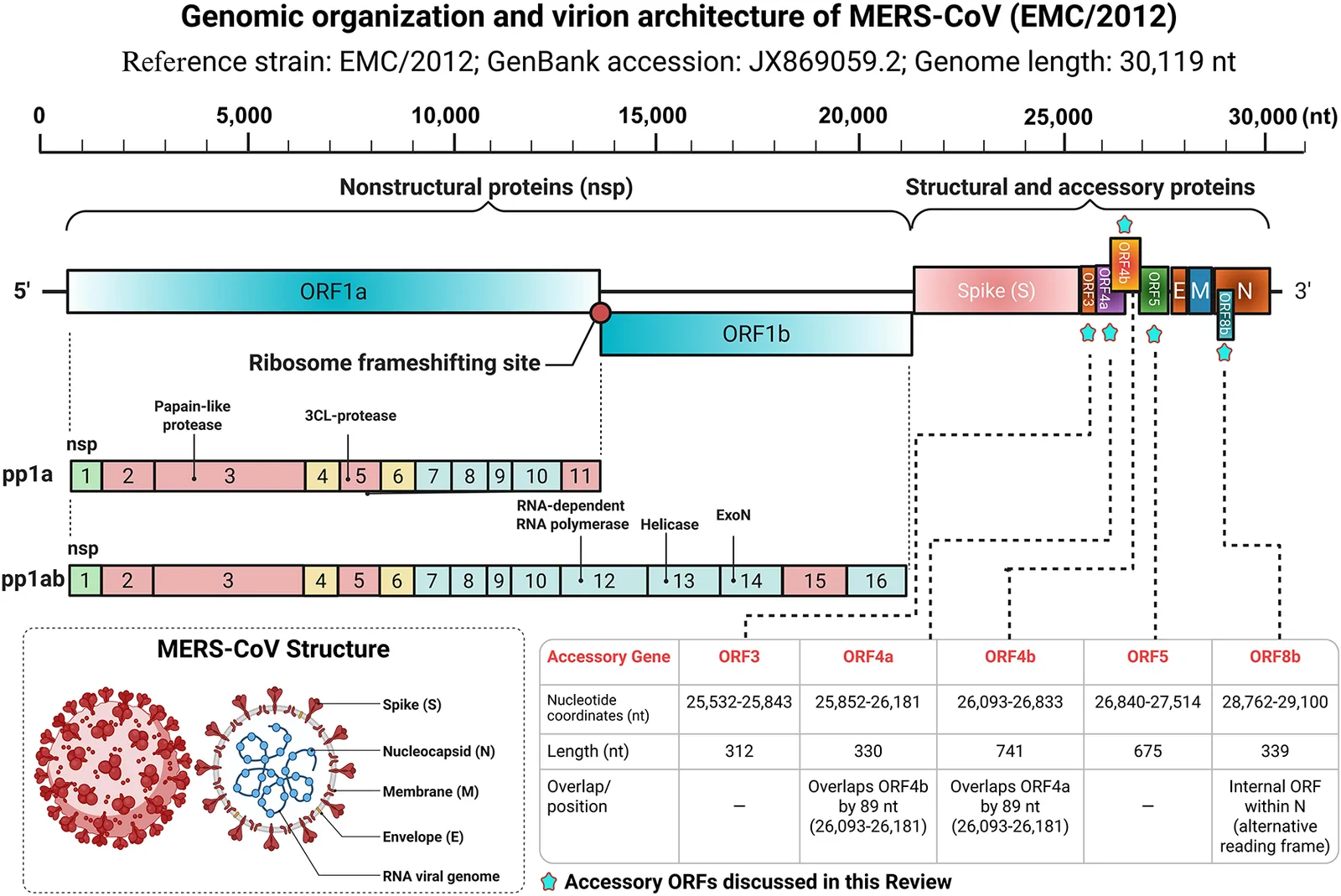
Genomic organization and virion architecture of MERS-CoV EMC/2012. The 30,119-nt EMC/2012 reference genome (GenBank JX869059.2) is drawn to scale and annotated with nucleotide coordinates for ORF3, ORF4a, ORF4b, ORF5, and ORF8b; ORF4a and ORF4b overlap by 89 nt, and ORF8b lies within N in an alternative reading frame (6). Blue star markers identify the accessory ORFs discussed in this Review; other colors distinguish genomic or virion components only. MERS-CoV, Middle East respiratory syndrome coronavirus; ORF, open reading frame; nt, nucleotide; nsp, nonstructural protein; pp1a/pp1ab, replicase polyproteins 1a/1ab; 3CL, 3C-like protease; ExoN, exonuclease; S, spike; E, envelope; M, membrane; N, nucleocapsid; RNA, ribonucleic acid.

### 3.2 Revising the concept of “dispensable proteins”: from accessory replicative factors to immune regulatory elements

In viral genetics, “dispensable” indicates that a gene is not absolutely required for a minimal replication cycle in a particular experimental system; it does not imply irrelevance to host responses or pathogenesis. Across coronaviruses, sequence-divergent accessory proteins repeatedly target interferon signaling, inflammation, cell death, and virus-host trafficking, illustrating functional convergence at the pathway level (8). Within the Merbecovirus lineage, ORF4b proteins from MERS-CoV and related bat viruses can localize to the nucleus and inhibit innate signaling (9). These two observations are conceptually distinct: the former concerns convergence across different coronaviruses, whereas the latter supports partial conservation of an ORF4b-associated phenotype within merbecoviruses. Neither observation establishes redundancy or synergy among MERS-CoV accessory ORFs.

### 3.3 From overexpression systems to reverse-genetics-based recombinant virus models

Research on MERS-CoV accessory proteins has progressed from ectopic-expression and reporter assays to reverse-genetics and infection models. Overexpression systems remain useful for screening localization, candidate interactors, and pathway effects, but they can exaggerate protein abundance and omit viral-genome context. Full-length infectious cDNA systems established that viruses lacking ORF5 or the ORF3–5 cluster can be recovered, although combined deletion can reduce replication and alter host responses (10, 11). Infection-level claims are therefore weighted most strongly when supported by authentic-virus genetics or *in vivo* experiments; infected-cell loss-of-function, primary-cell, and heterologous-virus studies provide model-bounded evidence; and ectopic-expression, reporter, and isolated biochemical studies establish mechanistic potential. **Table 1** applies this hierarchy to each research priority by pairing the supporting system and a quantitative or position-defined anchor with the principal inference boundary and next validation step.

**Table 1:** Evidence-graded research priorities for MERS-CoV accessory proteins.

Accessory protein/research priority	Evidence status/system	Quantitative or defined anchor	Required next validation	Key references
ORF4a: dsRNA binding, MDA5/PKR antagonism, and sequence-to-function evaluation	Mechanistically established; infection-level contribution supported by recombinant-virus and infected-cell studies; evolutionary significance unproven	EMC/2012 coordinates: nt 25,852–26,181; 89-nt ORF4a/ORF4b overlap. Comparative survey: n=568, described as highly conserved with greater SNP density in the terminal 45 nt, but no overall conservation percentage. Functional anchors: K63/K67 and ORF4a–PKR competition for viral dsRNA	Measure conservation and natural variation; test prioritized variants in matched viral backbones and host-relevant cells	Genome and variation (6):; dsRNA, MDA5, and PKR studies (15–20):
ORF4b: PDE domain, OAS–RNase L antagonism and nuclear-transport interface	Mechanistically established in biochemical, recombinant-virus, structural, and mouse-model studies; therapeutic relevance unvalidated	EMC/2012 coordinates: nt 26,093–26,833. Comparative survey: n=575; 13 African dromedary variants encoded 14–120-aa truncations. Functional anchors: PDE residue H182 and importin-binding residues R24/R33	Link natural variation to phenotype; evaluate selective inhibition, host toxicity, pharmacology, and infection-model efficacy	Genome and variation (6):; PDE (21):; KPNA4/importin interface (23, 24):; *in vivo* pathogenesis (27):
ORF5: background-dependent inflammatory modulation	Model- and backbone-dependent; supported by mouse reverse genetics and field-sequence analysis; not a unidirectional virulence determinant	Comparative dataset: 474/476 (99.5%) full-length ORF5 sequences; 60 substitutions (65% synonymous, 35% nonsynonymous) and two field deletions. Complete deletion produced a background-dependent pathogenesis phenotype	Analyze conservation and selection; test variants in matched viral backbones	ORF5 deletion and field-isolate analysis (33):
ORF8b: RLR/HSP70–IKKϵ signaling and model-dependent pathogenesis	Authentic-virus signaling and mouse-model evidence; pathogenesis direction differs between heterologous MHV and authentic mouse-adapted MERS-CoV	ORF8b coordinates: nt 28,762–29,100 within the N alternative reading frame. Residues 1–23 supported virulence enhancement in heterologous MHV, whereas ORF8b abrogation increased disease severity in mouse-adapted MERS-CoV.	Reconcile directional phenotypes in matched viral backgrounds using complementation, rescue, and quantitative virological and host-response readouts	Genome position (6); signaling (36); heterologous MHV (37); mouse-adapted MERS-CoV (38)
ORF3: HUWE1-regulated apoptosis and unresolved function	Cell-based HUWE1 mechanism; authentic-infection role unresolved. Population sequence associations are descriptive	Comparative survey: n=566; L17F/P86L in ~26% of camel-derived sequences (2014–2015) and ~93% of available human sequences (2015–2019); 9–41-nt deletions documented	Define ORF3 expression, localization, interactors, and function during authentic infection	Genome and variation (6):; HUWE1-regulated apoptosis (41):
Multi-accessory ORF deletion and attenuation	Authentic-virus evidence for a cluster-level phenotype; pairwise interaction terms unresolved	ΔORF3–5 attenuation contrasts with increased pathogenicity after ORF5 deletion and ORF8b abrogation in distinct mouse-adapted backgrounds	Perform combinatorial genetics, formal interaction analysis, complementation or rescue, stability and reversion testing, and cross-model validation	Accessory-ORF deletion and pathogenesis (33, 38, 42):; heterologous ORF8b context (37):
Human translational relevance	Pathway-level clinical concordance; ORF-specific causality unproven	Severe MERS has been associated with lower-respiratory IL-1/IL-8 and complement-related inflammatory signatures	Link viral genotype and accessory-ORF activity to longitudinal patient immune profiles	Human lower-respiratory inflammatory profiling (39, 40)

### 3.4 Evolution of accessory proteins in the merbecovirus context

Phylogenetic sampling supports bats as an evolutionary source of MERS-CoV-related viruses (12), while receptor studies demonstrate substantial variation in DPP4 use and the capacity of spike to adapt to species-specific DPP4 constraints (13, 14). These findings establish ecological and receptor diversity but do not show that accessory-ORF variation contributes to cross-species adaptation. A separate, untested working hypothesis is that, after receptor and entry barriers are met, accessory-ORF variation could modify intracellular fitness through host-specific differences in innate-immune antagonism. This possibility cannot be inferred from receptor-use data and requires matched viral backbones, defined naturally occurring variants, relevant cells from different host species, and infection-level endpoints. Accessory-ORF sequence variation should therefore be treated as a candidate layer for functional investigation rather than as an established determinant of host range or zoonotic transmission.

## 4 ORF4a: double-stranded RNA sensing, PKR stress responses, and interferon antagonism

### 4.1 ORF4a as an antagonist of the MDA5 pathway

ORF4a is among the best-characterized immune antagonists encoded by MERS-CoV. Niemeyer and colleagues showed that ORF4a weakens MDA5-mediated type I interferon induction (15) (**Figure 2**). Together with direct dsRNA-binding evidence (16), this work supports ligand sequestration as the principal explanation for reduced MDA5 access. The data position ORF4a near the RNA-sensing step; they do not show depletion of MDA5 or uniform blockade of all RNA sensors.

**Figure 2:**
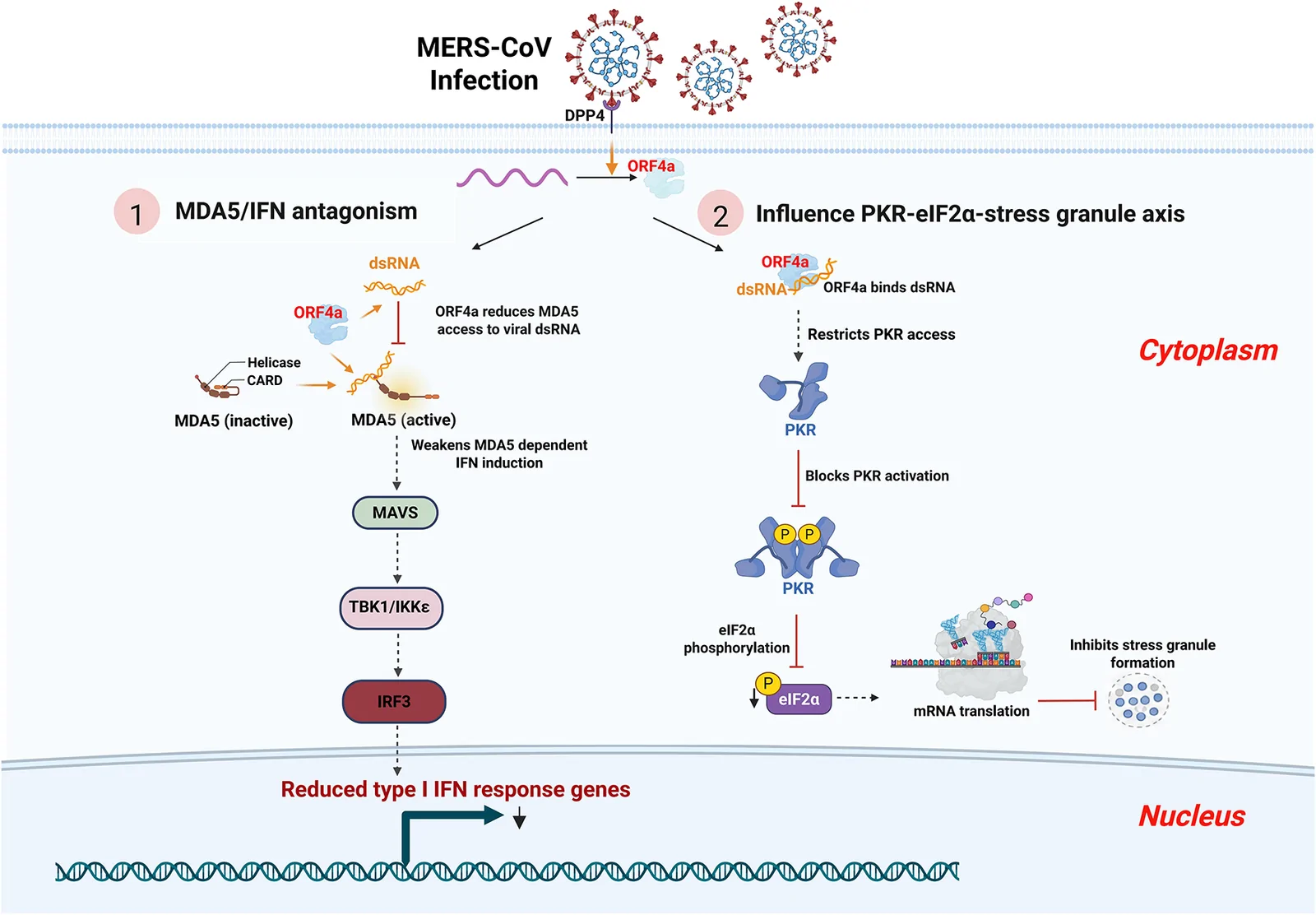
ORF4a antagonism of MDA5-dependent interferon induction and the PKR–eIF2α–stress-granule axis. ORF4a binds dsRNA and limits MDA5- and PKR-dependent responses in the represented experimental systems (15–20), including restriction of PKR access to dsRNA at replication organelles (17). Arrowheads indicate pathway direction or measured output; red blunt ends indicate inhibition; solid connectors denote relationships tested in the cited system, and dashed connectors provide pathway context. Yellow “P” symbols denote phosphorylation; other colors distinguish components only. MERS-CoV, Middle East respiratory syndrome coronavirus; DPP4, dipeptidyl peptidase 4; ORF, open reading frame; dsRNA, double-stranded RNA; MDA5, melanoma differentiation-associated protein 5; CARD, caspase activation and recruitment domain; IFN, interferon; MAVS, mitochondrial antiviral-signaling protein; TBK1, TANK-binding kinase 1; IKKϵ, inhibitor of nuclear factor kappa-B kinase subunit epsilon; IRF3, interferon regulatory factor 3; PKR, protein kinase R; eIF2α, eukaryotic initiation factor 2 alpha; mRNA, messenger RNA.

### 4.2 ORF4a suppresses the PKR–eIF2α–stress granule axis

PKR recognizes viral dsRNA and initiates translational arrest and stress-granule responses. Rabouw and colleagues showed that ORF4a binds dsRNA and blocks PKR activation, thereby reducing eIF2α phosphorylation and stress-granule formation (16) (**Figure 2**). A 2026 recombinant-virus study localized this interaction to the replication-organelle interface: PKR condensed on dsRNA exposed near double-membrane vesicles, whereas ORF4a competitively occupied viral dsRNA and restricted PKR access (17). Together, these studies define dsRNA shielding as the proximal mechanism; infection-level downstream phenotypes are compared below.

### 4.3 Functional convergence and combined-perturbation evidence during infection

Authentic-virus studies place ORF4a within a broader set of dsRNA antagonists. Comar and colleagues showed that ORF4a and ORF4b antagonize dsRNA-induced innate pathways during MERS-CoV infection, and later work tested EndoU in combination with ORF4a loss or ORF4b phosphodiesterase inactivation in lung and nasal epithelial cells (18, 19). The combined mutants enhanced dsRNA-responsive defenses and impaired replication in the tested systems, establishing joint EndoU–ORF4a and EndoU–ORF4b contributions. They do not establish pairwise ORF4a–ORF4b epistasis, additivity, synergy, redundancy, or hierarchy; those interaction classes require matched single and combined perturbations, rescue, and a prespecified quantitative expectation.

Downstream consequences remain cell dependent. In HeLa/CD26 cells, ORF4a deletion induced stress granules, while a virus lacking ORF4a and ORF4b showed PKR-dependent eIF2α phosphorylation and reduced viral translation (20). In A549-DPP4 cells, ORF4a deletion activated PKR without comparable eIF2α phosphorylation or translational arrest (18). ORF4a can therefore be placed at the dsRNA–PKR interface with greater confidence than any invariant downstream stress phenotype.

### 4.4 ORF4a variation as a candidate modifier of host-cell RNA-sensing thresholds

ORF4a dsRNA binding provides a defined mechanism through which natural sequence variation could alter MDA5- or PKR-dependent responses (15–17). This possibility remains untested as a determinant of host range or zoonotic transmission. In one comparative survey of 568 ORF4a sequences, ORF4a was described as highly conserved, with greater SNP density in the terminal 45 nt, but no overall conservation percentage was reported (6). K63 and K67 are experimentally defined dsRNA-binding residues (16), not validated markers of host adaptation. Sequence-to-function evaluation should therefore combine population-level conservation and variation with matched-backbone reverse genetics and infection in host-relevant cells (**Table 1**).

## 5 ORF4b: nuclear transport competition, NF-κB suppression, OAS–RNase L escape, and pulmonary inflammatory pathology

### 5.1 Phosphodiesterase activity of ORF4b and the OAS–RNase L pathway

ORF4b is one of the most structurally coherent and functionally complex MERS-CoV accessory proteins. Its N terminus contains a bipartite nuclear localization signal that drives nuclear entry, whereas its C terminus contains a phosphodiesterase-like domain that interferes with the OAS–RNase L pathway. The OAS–RNase L system is a key host defense mechanism that triggers RNA degradation upon detection of viral double-stranded RNA. Thornbrough and colleagues demonstrated that the PDE domain of the MERS-CoV ORF4b protein degrades 2′,5′-oligoadenylates required for RNase L activation, thereby limiting RNA cleavage after dsRNA sensing (21) (**Figure 3**). Yang and colleagues further showed that ORF4b acts on IKKϵ/TBK1-associated factors in the cytoplasm and suppresses type I interferon induction in the nucleus through its nuclear localization signal (22). ORF4a and ORF4b therefore converge on dsRNA-responsive defense at distinct checkpoints: ORF4a restricts ligand access to upstream sensors, whereas ORF4b limits downstream OAS–RNase L execution. This pathway juxtaposition does not demonstrate direct interaction between the proteins.

**Figure 3:**
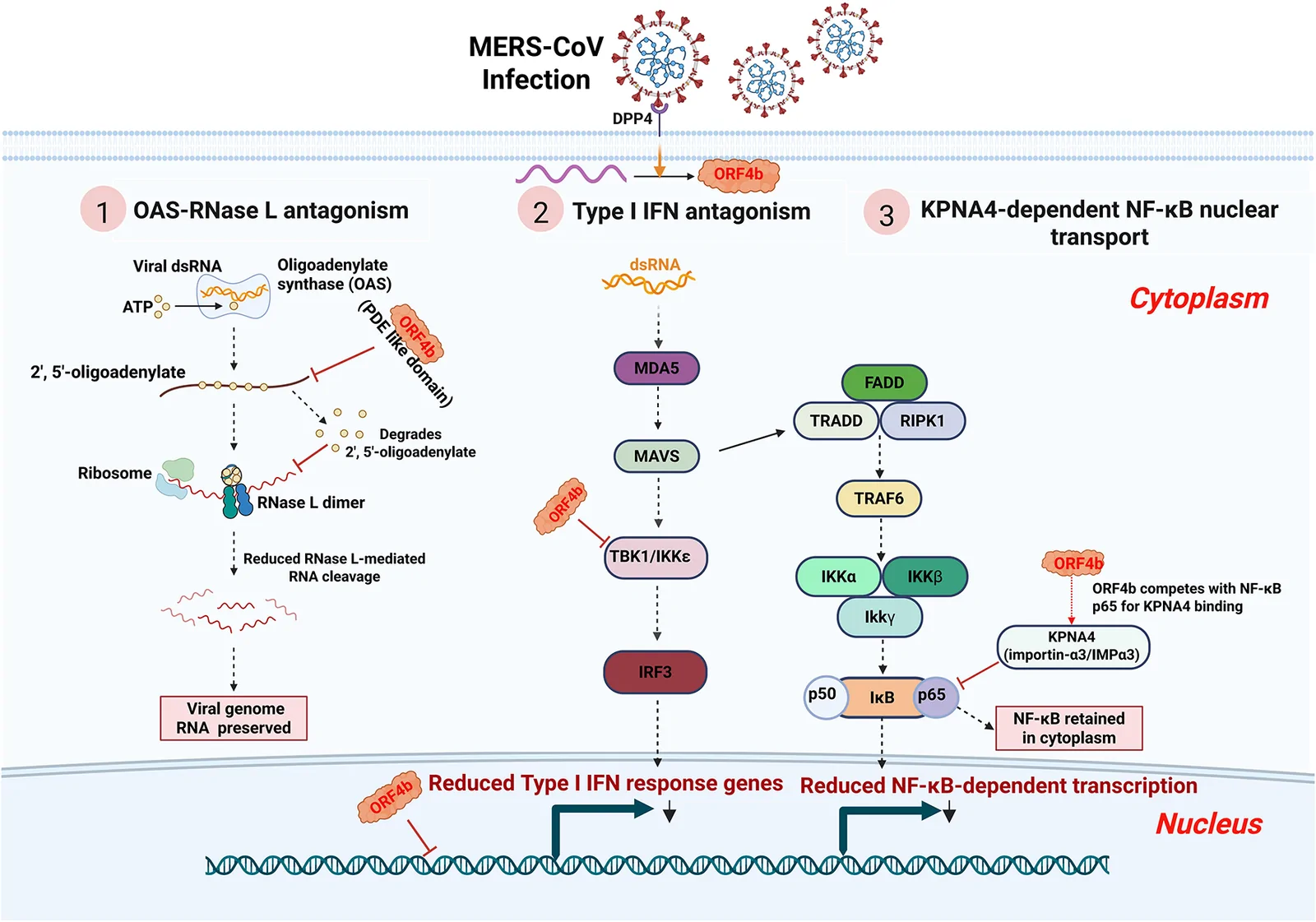
ORF4b antagonism of OAS–RNase L, type I interferon induction, and KPNA4-dependent NF-κB nuclear transport. The three panels summarize ORF4b phosphodiesterase-mediated 2-5A degradation (21), cytoplasmic and nuclear interferon antagonism (22), and competition with NF-κB p65 for KPNA4/importin-α3 (23, 24). Arrowheads indicate pathway or transport direction; red blunt ends indicate inhibition; connectors denote relationships tested in the cited system, and gray dashed connectors provide pathway context. Other colors distinguish components only. MERS-CoV, Middle East respiratory syndrome coronavirus; DPP4, dipeptidyl peptidase 4; ORF, open reading frame; dsRNA, double-stranded RNA; ATP, adenosine triphosphate; OAS, oligoadenylate synthetase; 2-5A, 2′,5′-oligoadenylates; PDE, phosphodiesterase; RNase L, ribonuclease L; MDA5, melanoma differentiation-associated protein 5; MAVS, mitochondrial antiviral-signaling protein; TBK1, TANK-binding kinase 1; IKKϵ, inhibitor of nuclear factor kappa-B kinase subunit epsilon; IRF3, interferon regulatory factor 3; IFN, interferon; KPNA4, karyopherin subunit alpha 4; IMPα3, importin alpha 3; NF-κB, nuclear factor kappa B; IKKα/IKKβ/IKKγ, inhibitor of nuclear factor kappa-B kinase alpha/beta/gamma; IκB, inhibitor of kappa B; p50/p65, NF-κB subunits.

### 5.2 ORF4b competitively blocks NF-κB nuclear transport through KPNA4

ORF4b nuclear entry is functionally linked to immune antagonism. In recombinant-virus infection, Canton and colleagues showed that ORF4b preferentially binds KPNA4 (karyopherin subunit alpha 4; also known as importin-α3/IMPα3), competes with NF-κB p65 for KPNA4 binding, and reduces NF-κB nuclear access (23) (**Figure 3**). The resulting change in inflammatory transcription is an infection-level mechanism that should be distinguished from ORF4b’s other cytoplasmic and nuclear effects.

### 5.3 Structural basis of the ORF4b–IMPα complex

Structural biology has provided an atomic-level explanation for ORF4b engagement of importin-α family members. Munasinghe and colleagues resolved ORF4b–IMPα complexes and showed that the ORF4b NLS occupies importin-α binding sites that overlap those used by NF-κB components (24). In the nomenclature used in that structural study, KPNA4 corresponds to IMPα3; this distinction is retained here to avoid conflating the KPNA gene number with the importin-α isoform name. Together with infection studies showing preferential interaction with KPNA4 (also known as importin-α3/IMPα3) and impaired NF-κB nuclear import (23), these findings define a structurally and functionally supported virus-host interface. This interface is a candidate for antiviral discovery, but selective inhibitors, pharmacology, host-toxicity profiles, and efficacy in MERS-CoV infection models have not yet been established; it should therefore be presented as a target-validation agenda rather than a clinically actionable therapeutic strategy.

### 5.4 ORF4b links innate signaling, protein stability, and inflammatory pathogenesis

ORF4b also regulates pathways beyond epithelial RLR signaling. In a TLR7/9 model, ORF4b bound IKKα and promoted its nuclear redistribution, impairing IKKα-mediated IRF7 phosphorylation and IFN-α induction (25). UBR5 provides a distinct host restriction mechanism by promoting K48-linked polyubiquitination and proteasomal degradation of ORF4b; UBR5 knockdown increased ORF4b-mediated immunosuppression and viral replication (26). In a mouse-adapted MERS-CoV/hDPP4-knock-in model, complete ORF4b deletion was fully attenuating and reduced pulmonary inflammatory pathology, whereas mutation of the ORF4b nuclear localization signal did not abolish lethality (27). Thus, the *in vivo* phenotype cannot be assigned solely to the KPNA4/NF-κB mechanism. Together with MERS-CoV combined-perturbation data (19) and coronavirus EndoU studies (28, 29), these results support convergence on dsRNA-responsive defenses but not a direct ORF4b–EndoU interaction.

### 5.5 Conservation, variation, and candidate surveillance relevance of ORF4b in merbecovirus

Genomic studies have resolved human transmission and regional circulation of MERS-CoV (30, 31), while outbreak sampling in dromedaries established the animal reservoir context (32). These findings justify monitoring accessory ORFs but do not show that an ORF4b variant predicts host range, transmission, or clinical outcome. A comparative survey analyzed 575 ORF4b sequences and identified 13 dromedary viruses from Nigeria, Morocco, and Burkina Faso (2015–2016) with deletions that encoded 14–120-aa truncations, as well as additional premature-stop or deletion variants in camel and human sequences (6). Functional anchors include the phosphodiesterase residue H182 (21) and the importin-binding residues R24 and R33 (24). Their natural variation remains hypothesis-generating until linked to epidemiological context and a reproducible phenotype in matched viral backbones (**Table 1**).

## 6 ORF5 and ORF8b: context-dependent inflammatory and interferon regulation

### 6.1 ORF5: from a small accessory protein to a context-dependent response modulator

ORF5 illustrates why MERS-CoV accessory proteins should not be assigned a single directional “virulence-factor” label. Early ectopic-expression studies suggested that ORF5 can modulate innate immune signaling by reducing IFN-β promoter activity and affecting IRF3/NF-κB-associated pathways (7). However, reverse-genetics analysis in a mouse-adapted MERS-CoV background revealed a more complex role: complete ORF5 deletion increased lethality compared with the parental virus, which already contained a 17-nt deletion and premature stop codon in ORF5, and resulted in delayed and dysregulated interferon and proinflammatory responses (33) (**Figure 4**). In addition, ORF5 showed high sequence stability among 476 analyzed human- and camel-derived field isolates (33). Collectively, these findings indicate that ORF5 is best viewed as a genetic-background-dependent modulator of host-response dynamics and pathogenesis rather than an intrinsically pro-virulent factor. The field-sequence result can be stated quantitatively: 474 of 476 sequences (99.5%) retained full-length ORF5; among these full-length sequences, 60 mutations were identified, of which 65% were synonymous and 35% nonsynonymous. Two field isolates contained ORF5 deletions—a 2-nt deletion in camel isolate D383/15 (KX108941) and a 19-nt deletion in human isolate Hu/Jeddah-KSA-3RS2702/2015 (KU851859) (33). These sequence data and the distinct reverse-genetics phenotype are compared explicitly in **Table 1** rather than being treated as equivalent evidence.

**Figure 4:**
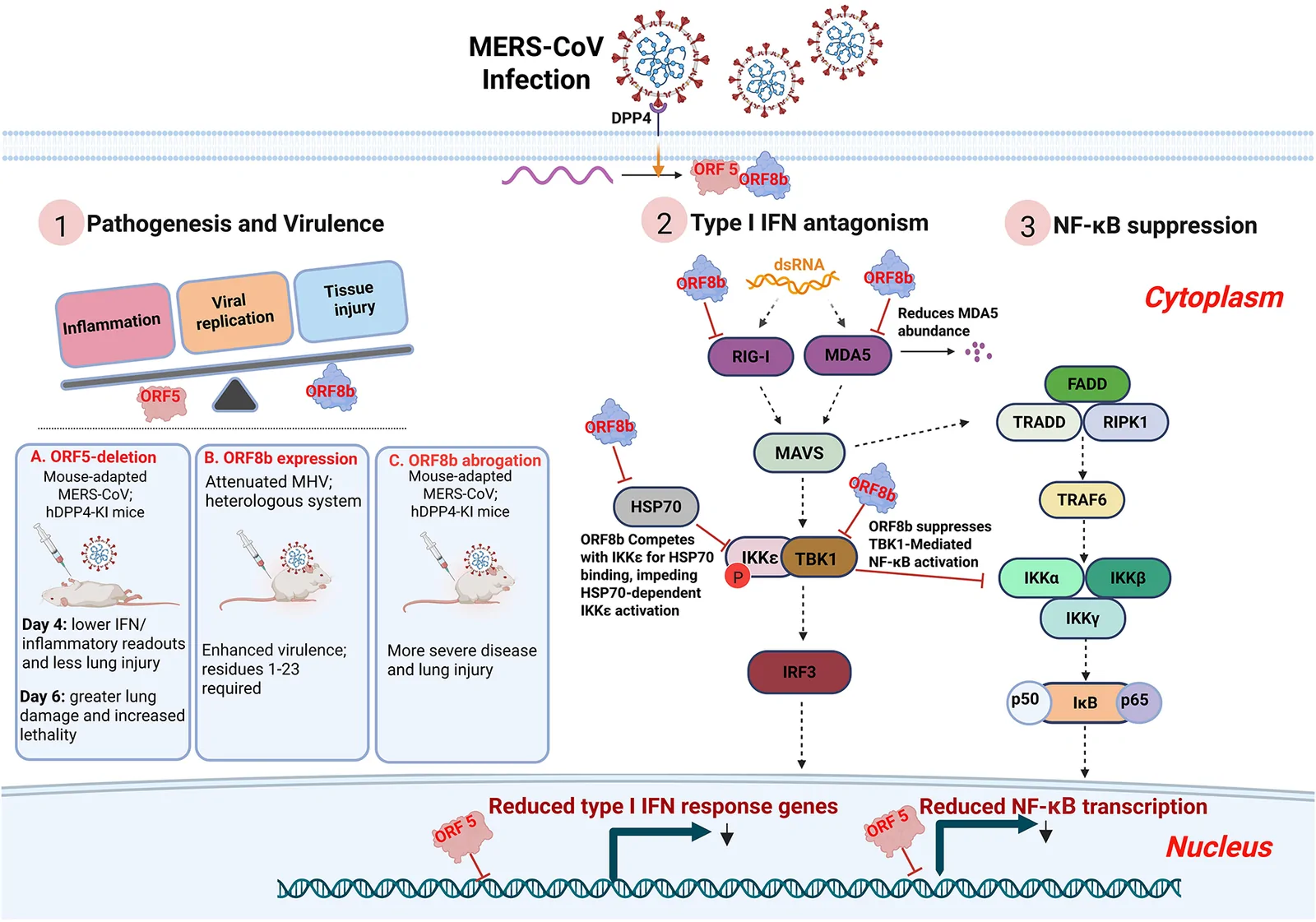
Context-dependent ORF5 and ORF8b functions. Panel 1 separates ORF5 deletion in mouse-adapted MERS-CoV (33), ORF8b expression in heterologous MHV (37), and ORF8b abrogation in mouse-adapted MERS-CoV (38). Panels 2 and 3 summarize interferon- and NF-κB-related mechanisms supported by ectopic-expression and infection studies (7, 34–36). These non-equivalent systems do not support a single directional virulence assignment. Arrowheads indicate pathway direction or measured output; red blunt ends indicate inhibition; solid connectors denote tested relationships, and dashed connectors provide pathway context. Colors distinguish components and model boxes only. MERS-CoV, Middle East respiratory syndrome coronavirus; DPP4, dipeptidyl peptidase 4; ORF, open reading frame; IFN, interferon; NF-κB, nuclear factor kappa B; dsRNA, double-stranded RNA; RIG-I, retinoic acid-inducible gene I; MDA5, melanoma differentiation-associated protein 5; MAVS, mitochondrial antiviral-signaling protein; HSP70, heat shock protein 70; IKKϵ, inhibitor of nuclear factor kappa-B kinase subunit epsilon; TBK1, TANK-binding kinase 1; IRF3, interferon regulatory factor 3; FADD, Fas-associated protein with death domain; TRADD, tumor necrosis factor receptor type 1-associated death domain protein; RIPK1, receptor-interacting serine/threonine-protein kinase 1; TRAF6, tumor necrosis factor receptor-associated factor 6; IKKα/IKKβ/IKKγ, inhibitor of nuclear factor kappa-B kinase alpha/beta/gamma; IκB, inhibitor of kappa B; p50/p65, NF-κB subunits; hDPP4-KI, human DPP4 knock-in; MHV, mouse hepatitis virus; aa, amino acids.

### 6.2 Model- and time-dependent ORF5 phenotypes

In hDPP4-knock-in mice infected with mouse-adapted MERS-CoV, complete ORF5 deletion was associated with lower IFN-β, inflammatory and ISG readouts, and less lung injury at day 4, but with more extensive lung damage at day 6. In MRC-5 human lung fibroblasts, the same deletion reduced IFN-β, ISG15, and proinflammatory transcripts despite similar replication kinetics (33). These temporally and experimentally distinct phenotypes support a role for ORF5 in regulating host-response kinetics rather than functioning as a fixed upstream inhibitory checkpoint.

These findings also explain why ectopic-expression and deletion phenotypes should not be placed on the same evidentiary footing. Reporter assays identify potential pathway effects, whereas reverse-genetics phenotypes integrate the complete viral background, host inflammatory environment, and sampling window. Matched-backbone infection studies are therefore required to determine whether ORF5 primarily alters replication, response timing, tissue injury, or the balance among these outcomes.

### 6.3 ORF8b suppresses RIG-I/MDA5-mediated IFN-β promoter activation

ORF8b antagonizes interferon signaling in several experimental systems. Ectopic-expression studies showed inhibition of RIG-I/MDA5-driven IFN-β promoter activation and effects on MDA5 abundance, while related reporter experiments found suppression of MDA5- and TBK1-mediated NF-κB activation (34, 35) (**Figure 4**). These assays establish mechanistic potential, not a uniformly acting upstream silencer; infection-level evidence is needed to define the relevant pathway and context.

### 6.4 ORF8b and the HSP70–IKKϵ–IRF3 axis

Mass spectrometry, co-immunoprecipitation, and recombinant-virus experiments showed that ORF8b is expressed during infection and suppresses IKKϵ/IRF3-dependent IFN-β induction by competing with IKKϵ for HSP70 binding (36) (**Figure 4**). An ORF8b-deficient virus increased IFN induction in infected Huh-7 cells, providing authentic-virus support for this mechanism. The signaling result does not predict whether deletion will attenuate disease *in vivo*; any attenuation strategy therefore requires matched-backbone tests of replication, immunopathology, genetic stability, and protective immunity.

At the pathway level, ORF8b acts at HSP70-dependent IKKϵ activation upstream of IRF3 (36), whereas ORF4a limits ligand access and ORF4b acts through distinct RNase L, kinase-associated, and nuclear-transport mechanisms (21–25). This represents convergence on interferon output, not evidence that ORF4b and ORF8b are redundant or operate in the same cellular context.

### 6.5 N-terminal determinants and model-dependent ORF8b pathogenesis

Evidence linking ORF8b to virulence requires especially careful qualification. Li and colleagues expressed MERS-CoV ORF8b in an attenuated murine coronavirus and found that ORF8b enhanced virulence in that heterologous viral background, with residues 1–23 required for the virulence-enhancing phenotype (37). The heterologous MHV experiment demonstrates virulence-enhancing potential, whereas genetic abrogation of ORF8b in authentic mouse-adapted MERS-CoV produced more severe disease, lung injury, and inflammatory-cell activation in hDPP4-knock-in mice (38). Considered with the authentic-virus HSP70–IKKϵ mechanism (36), these findings establish biological activity but not a universal direction of effect. **Table 1** therefore records residues 1–23 only as a heterologous-virus functional anchor and requires matched-backbone, complementation, and rescue studies before broader inference.

### 6.6 Human inflammatory signatures: translational concordance and limits

Human lower-respiratory-tract studies provide an important translational reference for evaluating whether inflammatory pathways implicated by MERS-CoV accessory-protein studies are observed during severe disease. Clinical analyses of MERS patients have identified increased pulmonary IL-1α, IL-1β and IL-8 together with altered inflammatory transcriptional responses (39). Independent investigations of severe or fatal cases reported increased complement-associated mediators, including C3a, C5a, factor P and RANTES, with several signatures associated with disease severity (40).

These clinical observations establish that inflammatory and innate-response pathways implicated by experimental MERS-CoV studies are active in human disease, but they do not identify which viral protein drives a given signature. Patient profiles integrate viral replication, structural and nonstructural proteins, host susceptibility, disease stage, treatment, and tissue injury. Because accessory-ORF genotype or activity has not been linked prospectively to longitudinal lower-respiratory immune profiles, the human evidence supports pathway-level concordance rather than ORF-specific causation.

## 7 ORF3 and multi-accessory-ORF deletion: from single-protein function to an evidence-graded systems framework

### 7.1 Limited evidence and unresolved questions for ORF3 function

Evidence for ORF3 remains limited compared with that for ORF4a and ORF4b. In transfected cells, ORF3 induced apoptosis in a dose-dependent manner, while HUWE1 promoted ORF3 ubiquitination and proteasomal degradation and reduced this activity (41) (**Figure 5**). Expression, localization, interaction partners, and contribution to disease have not been linked across authentic infection and *in vivo* models. A separate 566-sequence genomic analysis identified the L17F/P86L pair in about 26% of camel-derived ORF3 sequences from 2014–2015 and in almost 93% of the available human ORF3 sequences from 2015–2019, while also documenting 9–41-nt ORF3 deletions in some submissions (6). These are population-level temporal/host-source observations and do not establish an ORF3 adaptation mechanism; **Table 1** therefore keeps them separate from the HUWE1-regulated cell-based phenotype.

**Figure 5:**
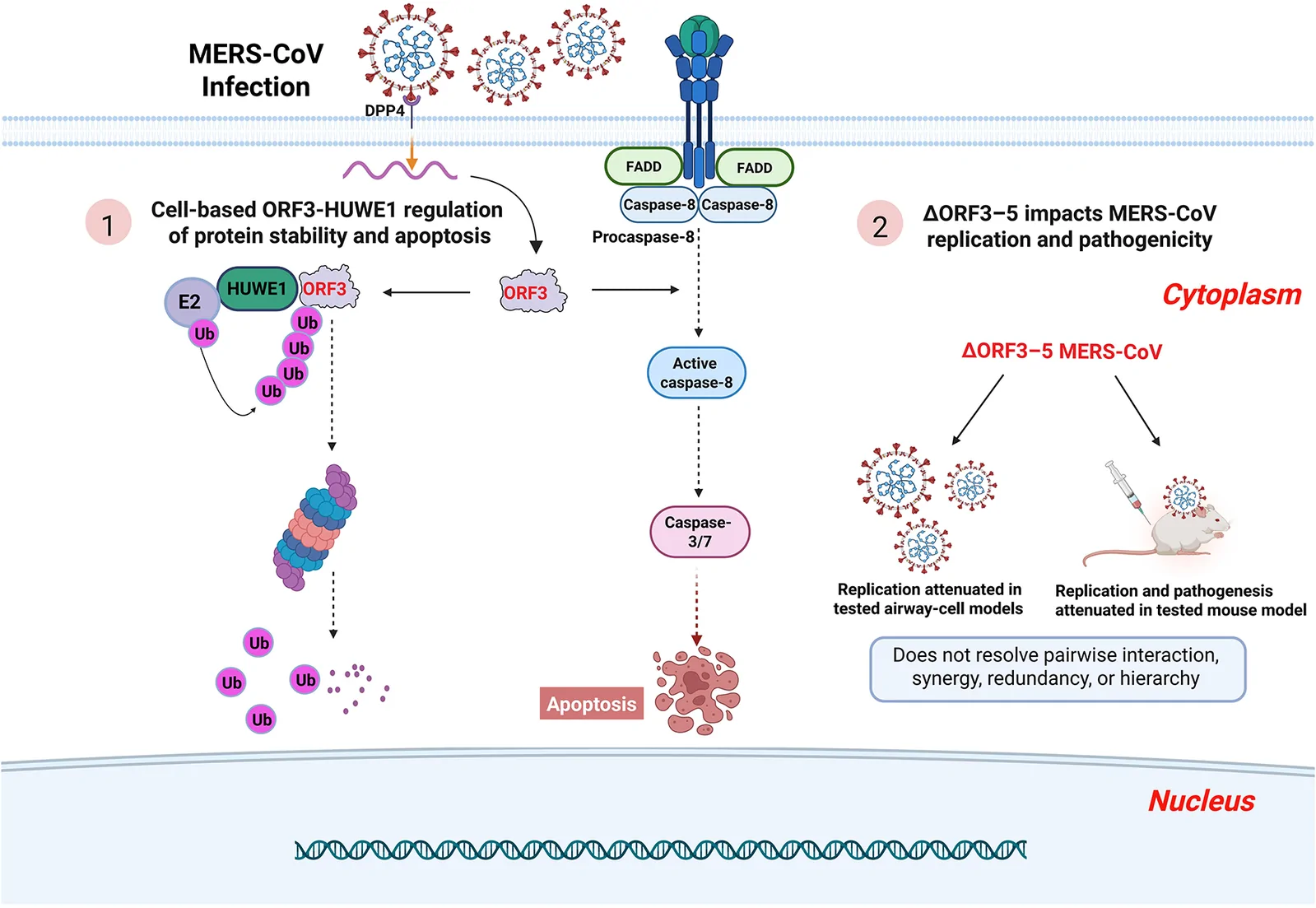
Cell-based ORF3–HUWE1 regulation and the cluster-level phenotype of ΔORF3–5 MERS-CoV. The left panel shows the ORF3–HUWE1 ubiquitin–proteasome/apoptosis mechanism established in transfected cells (41); its authentic-infection and *in vivo* roles remain unresolved. The right panel shows the replication and pathogenesis outcomes of the study-specific ΔORF3–5 virus (42); this combined deletion does not resolve pairwise interaction, synergy, redundancy, or hierarchy. Arrowheads indicate directional relationships or measured outputs; dashed connectors denote ectopic-expression relationships; colors distinguish components only. MERS-CoV, Middle East respiratory syndrome coronavirus; DPP4, dipeptidyl peptidase 4; ORF, open reading frame; ΔORF3–5, the study-specific combined deletion of ORF3, ORF4a, ORF4b, and ORF5; HUWE1, HECT, UBA and WWE domain-containing E3 ubiquitin-protein ligase 1; E2, ubiquitin-conjugating enzyme; Ub, ubiquitin; FADD, Fas-associated protein with death domain.

Because the ORF3–HUWE1 phenotype has not been mapped during authentic MERS-CoV infection, ORF3 cannot yet be placed confidently before or after the infection-level RNA-sensing, interferon, and inflammatory events assigned to ORF4a, ORF4b, ORF5, or ORF8b. In the integrated framework, ORF3 is therefore retained as a protein-homeostasis/cell-death branch with unresolved temporal and cell-type coupling to the other accessory proteins.

### 7.2 ΔORF3–5 mutant viruses provide cluster-level evidence for accessory-ORF function

Combined deletion of ORF3, ORF4a, ORF4b, and ORF5 provides the clearest infection-level evidence that the accessory-ORF cluster influences MERS-CoV phenotype. Menachery and colleagues showed that the ΔORF3–5 mutant has altered replication and pathogenesis, with attenuation driven largely by dysregulated host responses (42) (**Figure 5**). The phenotype establishes a causal contribution of the cluster but neither pairwise interaction nor formal synergy. **Table 1** therefore classifies ΔORF3–5 as cluster-level authentic-virus evidence; matched single and combinatorial deletions, complementation, and prespecified interaction models are still needed. The cluster phenotype is an integration anchor, not proof that all four ORFs act simultaneously or in a fixed order.

### 7.3 Integrating accessory proteins across pathway position, context, time, and genetic-interaction evidence

An integrated view emerges only when pathway position, cell or tissue context, sampling time, and genetic-interaction evidence are kept distinct. ORF4a is best supported at the dsRNA–MDA5/PKR interface (15–18, 20), whereas ORF4b acts at 2-5A–RNase L execution, kinase-associated interferon signaling, and KPNA4-dependent nuclear transport (21–24). ORF8b is positioned at HSP70-dependent IKKϵ activation, ORF5 at background- and time-dependent host-response regulation, and ORF3 at an incompletely validated protein-homeostasis and cell-death branch. Combined EndoU inactivation with ORF4a loss or ORF4b phosphodiesterase inactivation provides the strongest joint-perturbation evidence (19), but it does not resolve ORF4a–ORF4b epistasis or a universal hierarchy.

Cellular context and sampling time further constrain this synthesis. ORF4b can regulate TLR7/9–IKKα–IRF7 signaling relevant to IFN-α-producing immune-cell responses (25), whereas the ORF8b–HSP70–IKKϵ mechanism is supported during authentic infection of Huh-7 cells (36); these functions have not been shown to operate with equivalent weight in a single cell population. *In vivo*, ORF4b deletion reduces pulmonary chemokine expression and inflammatory pathology at 4–6 days post-infection in the tested mouse-adapted background (27), while complete ORF5 deletion produces a temporally different phenotype in which reduced early innate/inflammatory responses precede more severe later lung injury (33). ORF3 remains outside this temporal sequence because its HUWE1-regulated apoptotic phenotype is cell based (41). The resulting framework is therefore a matrix of tested contexts, not a sequential circuit or a claim that all accessory proteins act simultaneously.

Systems-level reviews identify recurrent coronavirus targeting of RNA sensing, interferon signaling, nuclear transport, translation, cell death, autophagy, and protein homeostasis (43–45). Comparative host–coronavirus interaction mapping likewise highlights shared host processes (46). Such convergence can organize mechanistic questions, but it does not establish equivalence among accessory proteins or a genetically hierarchical command layer.

Comparative epithelial studies show that the magnitude and timing of MERS-CoV-induced host responses depend strongly on experimental context. In Calu-3 cells infected at an MOI of 5, HCoV-EMC and SARS-CoV produced similarly limited transcriptomic changes before 12 h post-infection; at 18 and/or 24 h post-infection, 12,392 genes were differentially expressed after HCoV-EMC infection, of which 8,918 (72%) were regulated differently from SARS-CoV, demonstrating marked late transcriptomic divergence rather than a measurable global loss of innate signaling (47). In pooled-donor primary nasal air–liquid-interface cultures infected at an MOI of 1 and 33 °C, RNA sequencing at 96 h post-infection showed significant interferon-response upregulation after HCoV-229E, HCoV-NL63, and SARS-CoV-2 but not MERS-CoV; matched protein analysis showed no detectable increase above mock in STAT1 phosphorylation or representative interferon-stimulated-gene proteins after MERS-CoV infection, whereas HCoV-229E and HCoV-NL63 induced robust responses (48). Because the designs and endpoints differ, these observations remain specific to the tested models, inocula, temperatures, and time points.

## 8 Future strategies for mechanistic validation and translation

### 8.1 Mechanistic and therapeutic target validation

The ORF4a–dsRNA interaction (15, 16), ORF4b phosphodiesterase activity (21), ORF4b–KPNA4/importin-α interface (23, 24), and ORF8b–HSP70–IKKϵ axis (36) are experimentally defined virus-host interfaces that can guide target-discovery studies. The UBR5–ORF4b and HUWE1–ORF3 protein-stability axes (26, 41) provide additional mechanistic entry points, but host E3-ligase manipulation likewise requires direct evidence of selective target engagement and an acceptable host-safety profile. The translational status of all of these interfaces remains preclinical. There is not yet a validated set of selective inhibitors with demonstrated exposure, host safety, and efficacy against MERS-CoV infection for these accessory-protein interfaces. Future work should therefore progress from biochemical or structural screening to target-engagement assays, primary-airway models, recombinant-virus resistance mapping, and animal pharmacology before therapeutic claims are made.

### 8.2 Evidence-linked sequence-to-function evaluation

Sequence-to-function evaluation is most useful when population variation and experimentally defined function are kept separate. Published datasets provide sequence-scale anchors for ORF3 (n=566), ORF4a (n=568), ORF4b (n=575), and ORF5 (n=476), but these observations do not by themselves establish host adaptation or clinical relevance (6, 33). Variants should therefore be prioritized when they intersect experimentally defined functional anchors—such as ORF4a K63/K67 (16), ORF4b H182 (21), or ORF4b R24/R33 (24)—and then tested in matched viral backbones and host-relevant cells. ORF8b residues 1–23 remain a position-defined anchor from a heterologous MHV virulence assay rather than a validated surveillance marker (37). **Table 1** separates these data types and records the next causal test; without epidemiological linkage and infection-level evidence, accessory-ORF variation should not be interpreted as a marker of host range, transmission, or clinical risk.

### 8.3 Rational attenuation and cross-model safeguards

Reverse genetics demonstrates that accessory-ORF manipulation can alter replication, host responses, and pathogenesis, but these phenotypes define candidate attenuation hypotheses rather than a general vaccine-design rule. The ΔORF3–5 virus was attenuated in defined models (42), whereas complete ORF5 deletion increased lethality in one mouse-adapted background (33) and ORF8b abrogation increased disease severity in another (38). The directionally different ORF8b result in heterologous MHV (37) further illustrates model dependence. Candidate attenuated viruses therefore require matched-background assessment of replication, immunopathology, protection, epistasis, complementation or rescue, genetic stability, reversion or compensatory change, and phenotype across more than one relevant model (**Table 1**).

Model choice is central to this validation. hDPP4-transgenic and knock-in mice, mouse-adapted viruses, and other small-animal systems can define genetic effects and immune phenotypes (49–52), but species-specific receptor biology and adaptation mutations can modify accessory-gene phenotypes. More physiologically relevant airway cultures and, where justified, larger-animal models should be used to confirm findings that are intended to support translational decisions. The objective is not to construct a universal attenuation rule, but to establish which genetic changes remain predictably attenuating and immunogenic across controlled backgrounds and relevant hosts.

### 8.4 Cross-coronavirus comparison without mechanistic equivalence

Comparisons among SARS-CoV, SARS-CoV-2, and MERS-CoV reveal functional convergence despite marked sequence divergence (53), while *in vivo* studies show that proteins with related genomic placement or immune-antagonistic activity can have different effects on pathogenesis (38). Such comparisons can identify shared questions and contrasting solutions, but a mechanism or phenotype in one coronavirus should not be transferred to MERS-CoV without virus-specific evidence.

## 9 Discussion

Our review supports integration at the level of pathway architecture while showing that the genetic relationships among MERS-CoV accessory ORFs remain unresolved. ORF4a and ORF4b occupy the best-defined positions in dsRNA sensing, PKR stress responses, RNase L execution, and nuclear transport; ORF5, ORF8b, and ORF3 are more dependent on viral background, model, or evidence type. Combined EndoU–accessory perturbations and the ΔORF3–5 mutant provide joint and cluster-level causality, respectively (19, 42), but neither resolves the pairwise architecture among accessory ORFs. Taken together, the evidence reviewed here supports context-dependent functional convergence among MERS-CoV accessory proteins but does not establish a sequential regulatory circuit or fixed hierarchy.

Human lower-respiratory immune signatures provide an external test of biological plausibility but not ORF-specific attribution. Patient profiles integrate the effects of viral replication, structural and nonstructural proteins, host susceptibility, disease stage, treatment, and tissue injury; they cannot be used as proxies for the activity of an individual accessory protein. This boundary is important because pathway concordance between experimental models and severe disease does not establish that any one ORF determines clinical severity.

Several limitations constrain the present evidence base. Many mechanistic studies rely on ectopic expression, transformed cell lines, or single-pathway reporters; authentic-virus studies are fewer and often use different viral backbones, cell types, doses, and time points. Animal phenotypes can be strongly shaped by mouse adaptation and receptor engineering, as illustrated by the context-dependent ORF5 result. Evidence for ORF3 in authentic infection remains incomplete, ORF8b *in vivo* phenotypes are directionally discordant between heterologous MHV and authentic mouse-adapted MERS-CoV systems (37, 38), and natural-variation datasets rarely connect accessory-ORF genotype to transmission or clinical outcome. Similarly, structural druggability does not establish therapeutic selectivity, and deletion phenotypes do not by themselves establish vaccine safety. These limitations favor explicit evidence grading and matched-background validation over broad mechanistic generalization.

Future work should prioritize combinatorial reverse genetics that can formally test additivity, compensation, redundancy, and synergy; matched-backbone analysis of naturally occurring accessory-ORF variants; infection studies in primary human and relevant animal airway systems; and longitudinal linkage of viral genotype with lower-respiratory immune phenotypes in patients. Candidate antiviral interfaces should advance through target engagement, toxicity, pharmacokinetics, and infection-model efficacy, while attenuated-virus concepts require rigorous tests of genetic stability, reversion, immunopathology, and protection across models. These studies would make the framework quantitatively testable and clarify which accessory-protein effects are reproducible across MERS-CoV infection models.

## 10 Conclusions

MERS-CoV accessory ORFs constitute an experimentally uneven set of host-response modulators rather than a proven regulatory hierarchy. Evidence most securely places ORF4a at the dsRNA–MDA5/PKR interface and ORF4b at OAS–RNase L, nuclear transport, and model-bounded inflammatory regulation. ORF5 and ORF8b have causal but background-dependent phenotypes, whereas the ORF3–HUWE1 mechanism remains cell based. The ΔORF3–5 mutant establishes a cluster-level contribution without resolving additive, compensatory, redundant, or synergistic relationships among individual ORFs. The framework therefore defines the boundary between demonstrated accessory-protein functions and hypotheses that still require direct genetic testing.
